# Supplementary material for: Hock Lesions in Dairy Cows in Cubicle Housing Systems in Germany: Prevalence and Risk Factors
Source: Animals (Basel). 2023 Sep 14;13(18):2919. doi: 10.3390/ani13182919 (PMC10525860; doi:10.3390/ani13182919)
Supplement: Supplementary file 1 [file animals-13-02919-s001.zip › animals-2592292-supplementary.pdf]

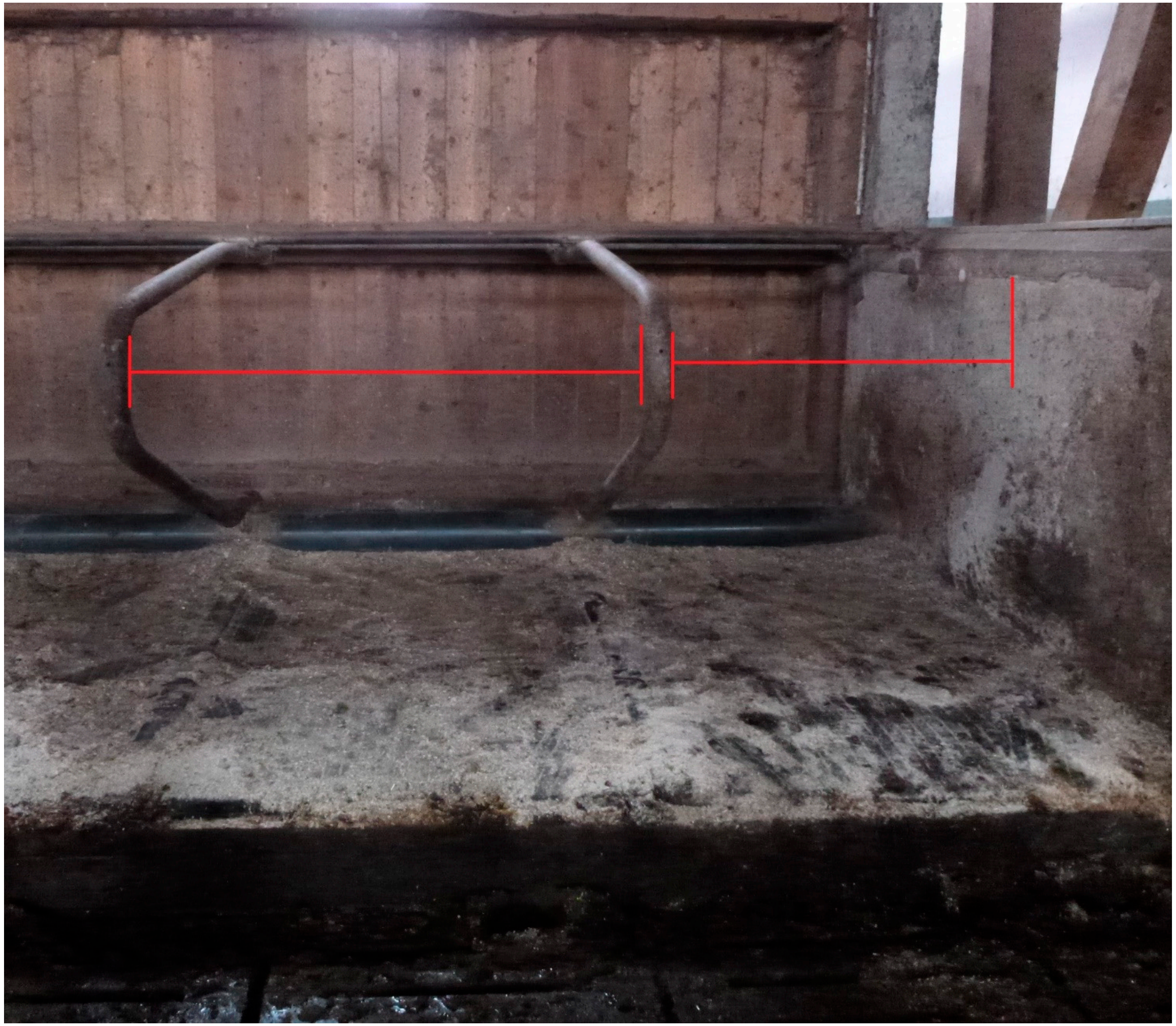

Figure S1. Measurement of the cubicle width for cubicles in the PraeRi-Study.

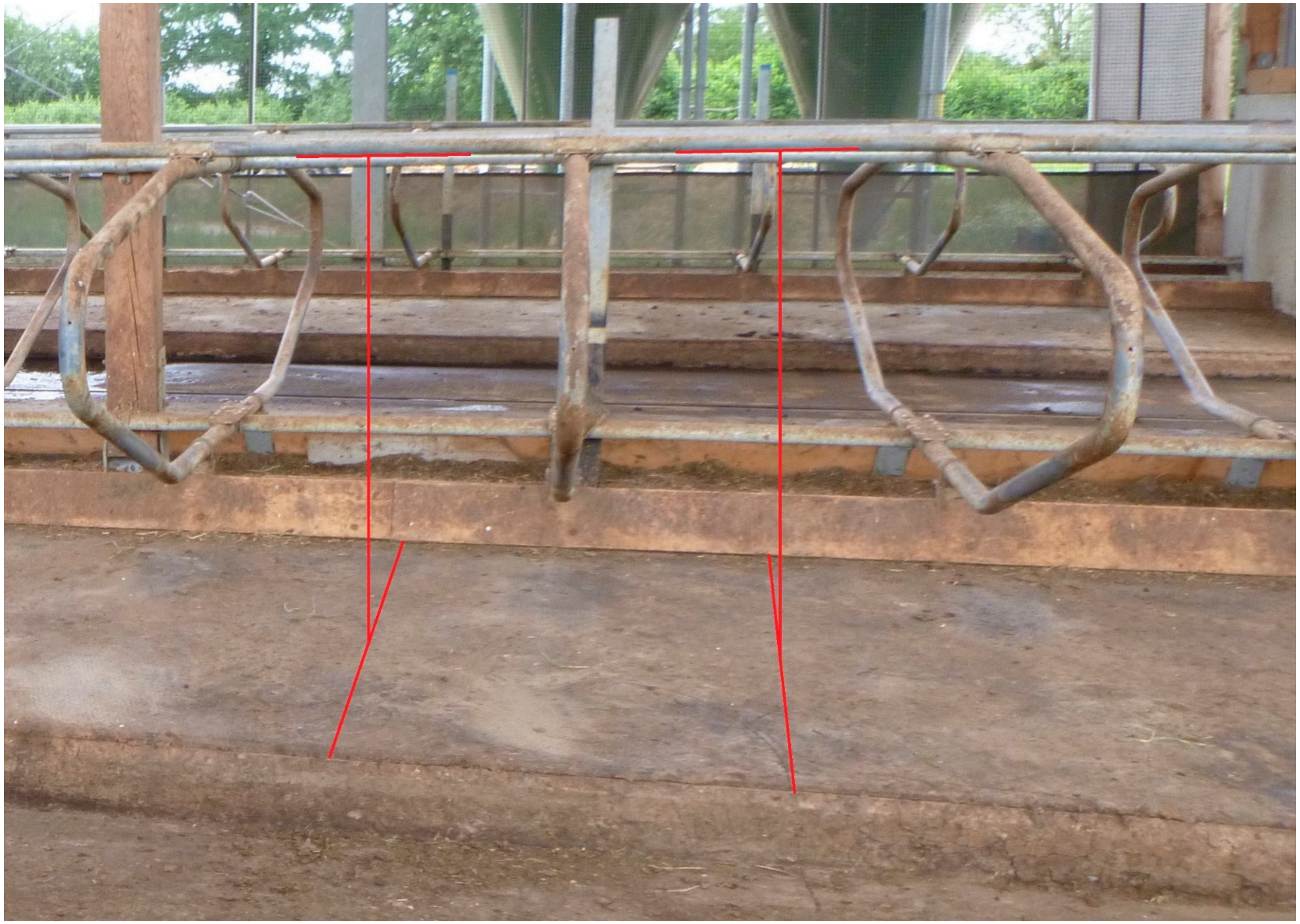

Figure S2. Measurement of neck bar height in cubicles in the PraeRi-Study.

Table S1. Hock lesions at the hock (HL) - Description at cow level of the categorical risk factors in the PraeRi-Study

Region		North				South				East			
Risk factor	Categories	no lesion		HL		no lesion		HL		no lesion		HL	
		n	%	n	%	n	%	n	%	n	%	n	%
Soiling of the lying surface	clean or single piles of faeces	2,688	25.6	7,823	74.4	1,549	38.3	2,500	61.7	7,990	31.1	17,719	68.9
	<50% of the surface soiled	2,184	31.2	4,817	68.8	960	36.2	1,695	63.8	1,867	20.8	7,119	79.2
	>50% of the surface or completely soiled	594	31.5	1,291	68.5	320	48.3	342	51.7	565	28.0	1,452	72.0
Bedding	no litter	387	12.9	2,618	87.1	217	11.7	1,637	88.3	1,125	11.8	8,430	88.2
	litter or only lime	3,115	21.3	11,535	78.7	2,579	47.3	2,871	52.7	9,313	34.3	17,824	65.7
Cubicle type	cubicles with or without a rubber mat	1,151	14.0	7,093	86.0	316	13.5	2,029	86.5	3,230	15.8	17,169	84.2
	deep bedded cubicles	3,900	47.1	4,380	52.9	2,371	55.6	1,874	44.4	6,737	45.1	8,202	54.9
	cubicles with comfort mattress	210	10.2	1,845	89.8	137	17.7	637	82.3	88	12.8	597	87.2
	other	241	22.4	834	77.6	5	62.5	3	37.5	381	54.7	316	45.3
Management of the farm	conventional	5,228	27.2	13,970	72.8	2,233	34.8	4,175	65.2	9,730	27.4	25,740	72.6
	organic or conversion	274	59.6	186	40.4	596	61.8	368	38.2	717	53.6	620	46.4
Grazing time	no grazing period	1,924	33.5	3,814	66.5	1,804	33.9	3,509	66.1	5,320	25.6	15,463	74.4
	0-2 hrs/day per animal	1,131	27.5	2,988	72.5	229	47.0	258	53.0	2,712	27.0	7,335	73.0
	2-4 hrs/day per animal	606	18.7	2,629	81.3	173	41.8	214	85.2	534	31.5	1,161	68.5
	> 4 hrs/day per animal	1,441	26.8	3,942	73.2	333	65.6	175	34.4	1,126	40.5	1,656	59.5
Median neck bar height > 125 cm	yes	2,048	21.9	7,306	78.1	698	26.4	1,949	73.6	4,115	26.6	11,369	73.4
	no	3,454	33.5	6,850	66.5	2,131	45.1	2,594	54.9	6,332	29.7	14,991	70.3
Median cubicle width > 115 cm	yes	1,651	31.8	3,535	68.2	1,994	41.8	2,776	58.2	2,577	33.9	5,017	66.1
	no	3,851	26.6	10,621	73.7	835	32.1	1,767	67.9	7,870	26.9	21,343	73.1
Lameness	normal gait (1)	3,085	34.7	5,803	65.3	1,537	48.9	1,606	51.1	3,309	41.9	4,583	58.1
	mildly lame (2)	1,309	25.7	3,783	74.3	800	35.5	1,452	64.5	3,609	27.9	9,313	72.1
	moderately lame (3)	804	20.0	3,220	80.0	334	27.9	865	72.1	2,438	23.9	7,769	76.1
	(severely) lame (4/5)	231	16.7	1,156	83.3	133	19.1	562	80.9	1,080	18.8	4,661	81.2
Age	< 3 years	1,587	34.7	2,989	65.3	632	45.4	759	54.6	3,854	33.1	7,790	66.9
	3-4 years	1,498	30.9	3,347	69.1	744	40.7	1,084	59.3	2,647	30.0	6,173	70.0
	4-5 years	995	26.5	2,766	73.5	491	36.2	865	63.8	1,731	26.6	4,783	73.4
	> 5 years	1,422	21.9	5,054	78.1	962	34.4	1,835	65.6	2,215	22.5	7,614	77.5

Table S2. Hock lesions (HL) - Description of the quantitative risk factors at cow level in the PraeRi-Study

Risk factor	Region		n	Mean	Median	STD *	CV *	Min	5% quantile	95% quantile	Max	Missing
Cow-cubicle-ratio	North	no HL	5,459	1.0	1.0	0.2	19.1	0.3	0.9	1.1	2.8	43
		HL	14,091	1.0	1.0	0.2	20.9	0.1	0.9	1.1	2.8	65
	South	no HL	2,829	1.0	1.0	0.2	17.1	0.3	0.9	1.1	1.7	0
		HL	4,540	1.0	1.0	0.2	19.3	0.2	0.9	1.2	2.0	3
	East	no HL	10,355	1.0	1.0	0.2	18.9	0.1	0.9	1.0	3.1	92
		HL	26,164	1.0	1.0	0.2	20.4	0.1	0.9	1.0	3.1	196
Body Condition	North	no HL	5,495	3.1	3.0	0.5	16.7	1.0	2.8	3.5	5.0	7
		HL	14,119	3.0	3.0	0.5	16.8	1.0	2.8	3.3	5.0	37
	South	no HL	2,824	3.7	3.8	0.5	13.6	1.0	3.5	4.0	5.0	5
		HL	4,538	3.7	3.8	0.5	14.5	1.3	3.5	4.0	5.0	5
	East	no HL	10,444	3.4	3.5	0.6	18.4	1.0	3.0	3.8	5.0	3
		HL	26,353	3.4	3.5	0.7	19.6	1.0	3.0	3.8	5.0	7
Lactation stage in days	North	no HL	5,323	201.7	186.0	137.6	68.2	0.0	91.0	291.0	800.0	179
		HL	13,812	211.6	199.0	132.1	62.5	0.0	107.0	297.0	800.0	344
	South	no HL	2,778	191.9	184.0	121.1	63.1	0.0	94.0	280.0	800.0	51
		HL	4,494	196.3	188.5	122.0	62.1	0.0	95.0	285.0	788.0	49
	East	no HL	9,828	181.6	163.0	126.1	69.4	0.0	80.0	263.0	800.0	619
		HL	24,707	201.5	191.0	127.5	63.3	0.0	100.0	283.0	800.0	1,653

* STD = standard deviation; CV = coefficient of variation

Table S3. Severe hock lesions (SHL) - Description of categorical risk factors at cow level in the PraeRi-Study

Region		North				South				East			
Risk factors	Categories	no SHL		SHL		no SHL		SHL		no SHL		SHL	
		n	%	n	%	n	%	n	%	n	%	n	%
Soiling of the lying surface	clean or single piles of faeces	9,008	85.7	1,503	14.3	3,552	87.7	497	12.6	20,874	81.2	4,835	18.8
	<50% of the surface polluted	5,961	85.1	1,040	14.9	2,320	87.4	335	12.6	6,675	74.3	2,311	25.7
	>50% of the surface or completely soiled	1,637	86.8	248	13.2	570	86.1	92	13.9	1,555	77.1	462	22.9
Bedding	no litter	2,327	77.4	678	22.6	1,408	75.1	466	24.9	6,191	64.4	3,424	35.6
	litter or only lime	14,493	87.0	2,157	13.0	4,991	91.6	459	8.4	22,961	84.6	4,176	15.4
Cubicle type	cubicles with or without a rubber mat	6,548	79.4	1,696	20.6	1,798	76.7	547	23.3	14,217	67.0	6,182	33.0
	deep bedded cubicles	7,727	93.3	555	6.7	4,035	95.0	210	5.0	13,810	92.4	1,129	7.6
	cubicles with comfort mattress	1,653	80.4	402	19.6	605	78.2	169	21.8	511	74.6	174	25.4
	Other	893	83.1	182	16.9	8	100	0	0.0	596	85.5	101	14.5
Management of the farm	conventional	16,378	85.3	2,820	14.7	5,535	86.4	873	13.6	27,988	79.0	7,482	21.0
	organic or conversion	444	96.5	16	3.5	911	94.5	53	5.5	1,209	90.4	128	9.6
Grazing time	no grazing period	5,016	87.4	722	12.6	4,531	85.3	782	14.7	16,299	78.4	4,484	21.6
	0-2 hrs/day per animal	3,491	84.7	628	15.3	456	93.6	31	6.4	8,088	80.5	1,959	19.5
	2-4 hrs/day per animal	2,685	83.0	550	17.0	353	91.2	34	8.8	1,276	75.3	419	24.7
	> 4 hrs/day per animal	4,597	85.4	786	14.6	481	94.7	27	5.3	2,153	77.4	629	22.6
Median neck bar height > 125 cm	yes	7,789	83.3	1,565	16.7	2,152	81.3	495	18.7	11,841	76.5	3,643	23.5
	no	9,033	87.7	1,271	12.3	4,294	90.9	431	9.1	17,356	81.4	3,967	18.6
Median cubicle width > 115 cm	yes	4,521	87.2	665	12.8	4,220	88.5	550	11.5	6,409	84.4	1,185	15.6
	no	12,301	85.0	2,171	15.0	2,226	85.5	376	14.5	22,788	78.0	6,425	22.0
Lameness	normal gait (1)	7,910	89.0	978	11.0	2,950	93.9	193	6.1	7,000	88.7	892	11.3
	mildly lame (2)	4,446	87.3	646	12.7	1,967	87.4	285	12.6	10,602	82.0	2,320	18.0
	moderately lame (3)	3,222	80.1	802	19.9	965	80.5	234	19.5	7,635	74.8	2,572	25.2
	(severely) lame (4/5)	1,018	73.4	369	36.6	490	70.5	205	29.5	3,926	68.4	1,815	31.6
Animal age	< 3 years	4,003	87.5	573	12.5	1,218	87.6	173	12.4	9,434	81.0	2,210	19.0
	3-4 years	4,219	87.1	626	12.9	1,620	88.61	208	11.4	7,130	80.8	1,690	19.2
	4-5 years	3,205	85.2	556	14.8	1,196	88.2	160	11.8	5,064	97.1	150	2.9
	> 5 years	5,395	83.3	1,081	16.7	2,412	86.2	385	13.8	7,569	77.0	2,260	23.0

Table S4. Severe hock lesions (SHL) - Description of the quantitative risk factors at cow level in the PraeRi-Study

Risk factors	Region		n	Mean	Median	STD *	CV *	Min	5% quantile	95% quantile	Max	Missing
Cow-cubicle ratio	North	no SHL	16,729	1.0	1.0	0.2	20.1	0.1	0.9	1.1	2.8	93
		SHL	2,821	1.0	1.0	0.2	22.7	0.1	0.9	1.1	2.8	15
	South	no SHL	6,443	1.0	1.0	0.2	18.5	0.2	0.9	1.1	2.0	3
		SHL	926	1.0	1.0	0.2	18.8	0.1	0.9	1.2	2.0	0
	East	no SHL	28,954	1.0	1.0	0.2	19.9	0.1	0.9	1.0	3.1	243
		SHL	7,565	1.0	1.0	0.2	20.2	0.1	0.9	1.0	2.4	45
Body Condition	North	no SHL	16,783	3.0	3.0	0.5	16.7	1.0	2.8	3.3	5.0	39
		SHL	2,831	3.0	3.0	0.5	17.0	1.5	2.8	3.3	5.0	5
	South	no SHL	6,438	3.7	3.8	0.5	13.8	1.0	3.5	4.0	5.0	8
		SHL	824	3.6	3.8	0.6	16.2	1.3	3.3	4.0	5.0	2
	East	no SHL	29,189	3.4	3.5	0.6	18.7	1.0	3.0	3.8	5.0	8
		SHL	7,608	3.3	3.3	0.7	21.2	1.1	2.8	3.8	5.0	2
Lactation stage	North	no SHL	16,364	207.2	195.0	134.1	64.7	0.0	100.0	294.0	800.0	458
		SHL	2,771	218.4	209.0	131.2	60.1	1.0	117.0	304.0	800.0	65
	South	no SHL	6,352	194.4	186.0	122.0	62.8	0.0	93.0	283.0	800.0	94
		SHL	920	196.5	191.0	119.0	60.6	2.0	100.0	278.0	717.0	6
	East	no SHL	27,430	192.8	180.0	127.4	66.1	0.0	90.0	274.0	800.0	1767
		SHL	7,105	207.7	196.0	126.5	60.9	0.0	110.0	289.0	800.0	505

* STD = standard deviation; CV = coefficient of variation
